# Enhanced nutritional value of mung bean microgreens compared to sprouts: a quantitative study

**DOI:** 10.1515/biol-2025-1278

**Published:** 2026-02-19

**Authors:** Sheba Sunny Marottickal, Neethu Saji, Noufan Valattil, Sudharsh Sajeev, Theertha Surendran, Ritty Kuriakose, Athira Syamala, Eva Ivanišová, Marcin Nowicki, Przemysław Łukasz Kowalczewski

**Affiliations:** Slovak University of Agriculture in Nitra, Faculty of Biotechnology and Food Sciences, Institute of Food Sciences, Nitra, 949 76, Slovakia; CSIR – Central Food Technological Research Institute Academy of Scientific and Innovative Research, Mysore, 570020, India; Quality, Health, Safety and Environment Officer at National Catering Services & Foodstuff, P.O. Box 39425, 904, Al Ferdous Tower, Salam Street, Abu Dhabi, United Arab Emirates; Department of Food Safety and Quality Assurance, Verghese Kurien Institute of Dairy and Food Technology, Kerala Veterinary and Animal Sciences University, Thrissur, Kerala, 680651, India; Slovak University of Agriculture in Nitra, Food Incubator, AgroBioTech Research Centre, Nitra, 949 01, Slovakia; Department of Entomology and Plant Pathology, University of Tennessee, Knoxville, TN, 37996, USA; Faculty of Health Sciences, Andrzej Frycz Modrzewski Krakow University, Kraków, Poland

**Keywords:** dietary fiber; functional foods; sustainability; *Vigna* *radiata*

## Abstract

Microgreens, the young edible seedlings of vegetables and herbs, have emerged as nutrient-rich alternatives in contemporary diets due to their enhanced sensory and nutritional attributes. This study investigates the nutritional composition and microbial quality of mung bean microgreens in comparison to mung bean sprouts. A consumer perception survey conducted via social media revealed that while awareness of microgreens’ health benefits is relatively high, actual consumption remains limited. On a fresh‑weight basis, microgreens showed lower protein and fat contents but nearly double the dietary fiber content compared to sprouts. Microgreens also exhibited elevated levels of essential minerals such as calcium, magnesium, iron, and zinc, with a slight reduction in potassium content. Furthermore, a significant increase in ascorbic acid (*p* < 0.05) was observed, whereas chlorophyll content showed no notable difference (*p* > 0.05). These findings suggest that mung bean microgreens may serve as a superior functional food component, offering a convenient, sustainable option to increase dietary fiber, selected minerals, and vitamin C in modern diets.

## 1 Introduction

In the semi-arid tropics of Asia, Africa, Southern Europe, and Central and Southern America, *Vigna radiata*, also referred to as mung beans or green gram, is a significant legume crop [1]. The Mung bean (*V. radiata* L.), alternatively known as the green gram, mash, moong, monggo, or munggo (Oxford Dictionary – Second Edition), is one of the most important edible legume crops, grown on more than six million hectares worldwide (about 8.5 % of the global pulse area) and consumed by most households in Asia [2]. Mungbean seeds are distributed for fresh use and the sprouts are the least expensive source of protein, calcium, phosphorus, and several vitamins [3]. Mung bean contains balanced nutrients, including protein, dietary fibre, minerals, vitamins, and significant amounts of bioactive compounds [4]. It is a rich source of protein (14.6 to 33.0 g/100 g) and iron (5.9 to 7.6 mg/100 g) and the anti-nutrients present are phytic acid, tannins, haemagglutinins and polyphenols. The mung bean induces less flatulence and is well tolerated by children [5]. Despite this, the presence of anti-nutritional factors in the mung bean may limit the biological value of its nutrients. For example, phytic acid can bind to several important divalent cations such as iron, zinc, calcium, and magnesium. The insoluble complexes formed in result of this binding can limit the mineral absorption and utilization in the small intestine [6]. Wang et al. [7] reported that after germination, the phytic acid contents declined in mung beans by 76 %, and bioavailability values of zinc and iron increased were 3.0 and 2.4 times higher than that of raw mung beans, respectively. Mungbean is a highly nutritious and easily digested grain legume with a greater range of adaptability, a shorter crop length, and soil-improving qualities. Mungbean seeds’ overall nutritional quality is improved by sprouting, which lowers antinutritional elements and raises the seeds’ economic value [8]. Mungbean seeds are sprouted for fresh use or canned for shipment to restaurants [9]. Cooks, Culinologists, and consumers should continue to hail beans as a vital food because of their great adaptability and versatility. It is anticipated that the bean’s culinary importance will continue to grow as consumers increasingly strive for healthier diets. Products and ingredients based on beans and pulses present prospects for new products aimed at retail and foodservice consumers [10]. According to European Union (EU) Regulation (EC) No 208/2013, Sprouts are “the product obtained from the germination of seeds and their development in water or another medium, harvested before the development of true leaves and which is intended to be eaten whole, including the seed” [11]. Vegetable foods like sprouts are high in phytonutrients such isoflavones, phenolics, and glucosinolates. Sprouts are also rich in vitamins and minerals, according to numerous research. Sprouts can show a decrease in anti-nutritional elements including phytates, tannins, and oxalates in addition to their high nutrient concentration, which improves the minerals’ bio-accessibility. However, a number of variables, like the kind of sprout and the germination conditions, affect their nutritional makeup [12]. Sprouts are high in protein (21–28 %), calcium, phosphorus and certain vitamins. Because they are easily digested, they replace scarce animal protein in human diets in tropical areas of the world [9]. During sprouting, the proteolytic cleavage of these proteins, amino acids, vitamins, and minerals are significantly high [13]. Mung bean sprout production is a simple germination process that requires neither sunlight nor soil; it has no season limitations. Mung bean seeds that have germinated have a substantial potential for low-temperature waste heat [14]. The sprout production is extremely inexpensive, requiring only mung bean seeds, sprouting containers and water as inputs. It can, therefore, be practiced even by poor farmers in augmenting their meager resources. Beans stored under 0 °C and 85 % relative humidity produce good quality sprouts. Seeds with 15 % moisture can be safely stored for one year at 10 °C or below [15]. The sprouting period is typically 5 days but is dependent on the sprouting temperature applied [16]. Because of their major use as sprouts, a high-quality seed with excellent germination is required. The food industry likes to obtain about 9 or 10 g of fresh sprouts for each gram of seed [9]. Mung bean and sprouts are widely consumed as a fresh salad, vegetable or even as a common food in India, China, Bangladesh, Philippines, Thailand, Southeast Asia, and western countries [17]. People in countries like China, Japan, India, and others have been eating mung and adzuki bean sprouts for generations as part of their traditional food products because of its high nutritional content. Phytic acid and other antinutritional elements in beans can be lessened by the physicochemical changes that occur during sprouting. Adzuki beans and sprouted mung have better digestion and a higher total nutritional value [18].

Microgreens (MG) are distinct from sprouts even if both greens are consumed in an immature state [19]. They are a new class of edible vegetables [20], a very specific type which includes seedlings of edible vegetables, herbs or other plants, ranging in size from 5 to 10 cm. Harvested seven to fourteen days after germination, microgreens are immature vegetable greens that provide concentrated nutrients and bioactive substances [21]. Depending on the species that has been used, they can be harvested 7–21 days after germination when the cotyledonary leaves have fully developed and the first true leaves have emerged [22]. It is mainly cultivated in East Asia, Southeast Asia and the Indian subcontinent. Despite small in size, microgreens, also known as “vegetable confetti” [19] or “microherbs” when referring to aromatic herbs, can provide a wide variety of intense flavours, bright colours and a good texture; therefore, they may be proposed as a new ingredient to enhance and garnish drinks, salads, appetizers, main and second courses, soups, sandwiches and desserts [19], 22]. Phytonutrient levels differ according to the growth stages of the plant and often decrease from the seedling (sprout, microgreen) to the fully developed stage [23], [24], [25], [26]. In addition to their high nutritional value, microgreens are considered functional foods with health-promoting or disease preventing properties [27]. Because they are created and chopped down before ripening, microgreens contain vital bioactive elements that operate as functional meals. Since these are the tiny cotyledons of various pulses, grains, and vegetable seeds, their nutritional profile differs from that of the whole one, which contains essential fatty acids, the main one being linoleic acid, and functional compounds such as glucoraphanin, which signals the antioxidants, glucosinolates, isothiocyanates, anthocyanins, ascorbate peroxidase, catalase, superoxide dismutase, and peroxidase, which fight oxidative stress and inflammation and, in turn, diabetes, and cancer [28]. Glucosinolates, phenolics, flavonoids, vitamins, minerals, and pigments are some of the health-promoting substances found in microgreens that demonstrate their potential as a nutritional powerhouse [29]. Supplementation with microgreens attenuated body weight gain, lowered low-density lipoproteins (LDL) cholesterol levels, reduced hepatic cholesterol ester and triglyceride levels, and inflammatory cytokines. Supplementation of low-fat diet with red cabbage microgreens raised both low-density lipoprotein and high-density lipoprotein cholesterol levels [30]. Microgreens are prized for their vivid colours, concentrated flavours, clean, soft texture, and tightly packed nutrients. Due to their strong flavours, enticing sensory attributes, practicality, and profusion of vitamins, minerals, and other bioactive substances like ascorbic acid, tocopherol, carotenoids, folate, tocotrienols, phylloquinones, anthocyanins, glucosinolates, etc., microgreens have become increasingly popular among high-end restaurant chefs and nutritional researchers in recent years. These characteristics drew interest from researchers for application in the fields of nutrition and human health [31]. Microgreens are 4–6 times more nutrient dense than their mature counterparts [22]. When taken as dietary supplements, microgreens, which are part of a class of functional foods, offer a variety of health advantages and important nutritional components. Its presence of various health-promoting components has led to a dramatic increase in consumption compared to their mature plants [32]. Microgreens are ideal for space flight environments as they can be harvested directly by crew members, ensuring freshness and high quality. Their production can be implemented on static, shallow substrates with little or no nutrient supplementation, and this alleviates problems of poor crop performance associated with low O2 and nutrient solubility in microgravity hydroponic systems [33]. Space agencies have also shown interest in microgreens, believing that their sensory properties might supplement astronauts’ diets in microgravity and that growing them could support crew members’ physical and mental well-being during extended spaceflight missions [34]. Mung bean microgreens represent a candidate species for such applications, combining rapid production with high fiber and mineral contents.

So far, sprouts have been more often than microgreens recognized as wellness and health-promoting foods, widely recommended by dietitians due to their high content of nutrients and bioactive compounds, such as flavonoids, hydroxycinnamic acids, vitamins and glucosinolates, minerals, and carotenoids [35], 36]. However, microgreens have much stronger flavour enhancing properties than sprouts, and a broad range of leaf colour, variety and shape [37]. Among the notable functional foods, microgreens have the potential to improve lifespan and health. Though microgreens do not require much care after the spreading of seeds, sufficient moisture should be maintained through fine spray. High light requirement of 12–16 h of period should preferably be maintained along with low humidity and good air circulation for better growth and development of microgreens [38]. In the twenty-first century, microgreens have gained popularity as a salad-style food that can meet certain nutrient needs. Microgreens are young seedlings that offer a wide range of colours, flavours, and textures. Because of their nutraceutical qualities, they are referred to as a “functional food.” Microgreens are a promising option for producing high-value crops, and growing these nutritious horticultural crops requires optimising cultural practices such as seed density, growing media, irrigation water safety, light quality and quantity, temperature, and relative humidity [39].The appeal of microgreens to consumers, coupled to their high price market and short production cycle, has attracted greenhouse growers and many urban and peri-urban farms have invested in their production. Products made from fresh and processed microgreens can improve dietary variety, nutritional value, and appeal [40]. On the other hand, microgreens low yield, rapid senescence and very short shelf-life curb the expansion of their commercial production [41], 42]. Despite having the potential to develop into a useful food in the future, the microgreens sector is unable to meet population demands in terms of yield. Reduced production and shorter shelf life are two significant challenges the microgreen sector faces that need for extra care. The agriculture sector has also embraced several techniques, such as hydroponics, aeroponics, and aquaponics, in addition to nanotechnology, to produce microgreens that are more nutritious, high-yielding, and require less energy [43].

Sprouts and microgreens can be efficiently produced in urban and peri-urban environments, where land availability is limited, either by specialized growers or consumers themselves. Unlike sprouts, the term “microgreens” is a marketing designation rather than a scientific classification [44]. Their cultivation began in the late 1980s [35], 36] and has since gained momentum, driven by increasing consumer interest in functional foods. Nutrient-dense sprouts and microgreens can be produced with little input because of their short development cycle; they can even be grown at home and picked as needed without the use of pesticides, resulting in minimal environmental effect and widespread appeal among health-conscious consumers [45]. Microgreens and sprouts are characterized by low calorific values (29–128 kcal/100 g) and low glycaemic indices [35], 46] Young, delicate leafy vegetables known as microgreens are becoming more popular among consumers. This is explained by their high antioxidant and micronutrient content and low calorie content [47]. Microgreens are rich in ascorbic acid, chlorophylls, carotenoids, and essential micronutrients such as potassium, magnesium, copper, iron, and zinc. Before they reach full maturity, microgreens are harvested shortly after they sprout. Legume microgreens, such as lentils and chickpeas, have a high nutritional value and provide a concentrated source of nutrients. In addition to vitamins A, C, and K, legume microgreens include iron, calcium, and other minerals. They also possess antioxidants, which aid in their defence against the damaging effects of free radical damage [48]. Compared to sprouts, microgreens exhibit stronger anti-diabetic and anticholinergic properties. Owing to their high vitamin content and low carbohydrate and anti-nutrient levels, microgreens are increasingly recommended as dietary supplements, particularly for individuals seeking low-carbohydrate nutritional options. Moreover, they contribute to enhancing the flavor and sensory quality of foods [49]. The extraction, isolation, and characterization of bioactive compounds from microgreens is an emerging area of research, particularly within biomedical and related sectors [29]. When compared to microgreens produced in soilless media, those cultivated in soil had a higher antinutrient content. For the production of microgreens, soilless medium offers a viable and sustainable solution, particularly in urban areas with limited resources. The production and quality of microgreens can be improved by optimising the growing medium, which will help the microgreens sector expand and support sustainable food systems [50]. Despite being cost-effective and relatively simple to cultivate, mung bean microgreens remain under-investigated in terms of their complete nutritional profile [51]. Recent years have seen a great deal of research on sprouts and microgreens, but there are still a number of undiscovered or understudied elements. The production stage (e.g., genotypic analyses, seed treatments, elicitation techniques, biofortification), post-harvest processing (e.g., seed sanitation, shelf-life extension), the effects on human nutrition and animal feeding, and, most intriguingly, drug discovery are all potential areas for future research [52]. Limited evidence suggests that young seedlings can possess significantly higher concentrations of vitamins, minerals, and phytonutrients than their mature counterparts [20], 53]. Within this context, the present study gains relevance by exploring mung bean microgreens as a sustainable and nutrient-dense alternative for the modern diet in comparison with the sprouts.

## 2 Materials and methods

### 2.1 Survey

As part of this study, data were collected using the Computer-Assisted Web Interviewing (CAWI) method. A structured questionnaire was developed and implemented via the Google Forms platform. The survey link was disseminated through multiple social media channels to ensure broad public outreach. This approach enabled the efficient gathering of responses from a diverse sample. The collected data provided valuable insights into public awareness, usage patterns, and perceived health benefits associated with microgreens.

### 2.2 Production of sprouts and microgreens

Mung bean seeds were sourced from local markets in Mannuthy, Thrissur, Kerala, India. The seeds were thoroughly washed with potable water and soaked for 12 h at room temperature (27 ± 2 °C) to initiate germination. After soaking, the seeds were evenly spread in a single layer on tissue paper within plastic trays. The trays were watered twice daily with potable water to facilitate the sprouting process. Once germination occurred, the sprouts were maintained under optimal conditions near a window to receive adequate sunlight, and watering continued twice daily for 7 days at room temperature (27 ± 2 °C).

Following the sprout stage, a separate set of seeds was allowed to grow further into microgreens. These sprouts were transitioned into the microgreen growth phase by continuing to water them twice daily for another 7 days, until the first true leaves emerged. The microgreens were monitored for the development and growth of their true leaves over this 14-day period to assess nutritional and morphological changes.

### 2.3 Determination of moisture content

Moisture content of microgreens, sprouts, and raw mung bean pulse samples was determined using the Association of Official Analytical Chemists (AOAC) gravimetric method (AOAC, 1990) [54]. Briefly, approximately 5 g of sample was oven-dried at 105 °C to constant weight, and moisture content was calculated as the percentage weight loss during drying.

### 2.4 Determination of crude protein

The crude protein content of microgreens, sprouts, and raw mung bean pulses was determined using the Kjeldahl digestion followed by distillation method [55]. Protein content was calculated by multiplying the percentage of nitrogen by a factor of 6.25, based on the assumption that most proteins contain approximately 16 % nitrogen. For sample preparation, 0.5 g of the mung bean sample was finely crushed using a mortar and pestle. This sample was then mixed with 10 mL of concentrated H_2_SO_4_ in a digestion flask, followed by the addition of 2 g of the catalytic mixture. The sample was digested for 4 h. After digestion, the digest was diluted to a final volume of 100 mL using a volumetric flask and was used for subsequent analysis. A 10 mL aliquot of the digest was then combined with an equal volume of 40 % NaOH solution in a Kjeldahl distillation apparatus. The mixture was distilled into 20 mL of 4 % boric acid solution containing three drops of a mixed indicator. A total of 50 mL of distillate was collected and titrated against 0.1 N HCl, with the endpoint being reached when the color changed from green to deep red. A blank sample was processed in the same manner as the test samples.

### 2.5 Determination of crude fat

Crude fat was determined for microgreens, sprouts as well as raw pulse of mung beans. This was determined by solvent extraction gravimetric method described by Kirk and Sawyer [56], and AOAC (1984) [57]. The sample was prepared by drying it in the hot air oven at 100 °C for 1 h. 10 g of the sample was wrapped in a porous paper (Whatman filter paper) and put in a thimble. The thimble was put in a Soxhlet Reflux Flask and mounted into a weighed extraction flask containing 200 mL of diethyl ether. The upper part of the reflux flask was connected to a water condenser. The solvent (diethyl ether) was heated, boiled, vaporized and then condensed into the reflux flask and filled in. Soon the sample in the thimble was covered with the solvent until the reflux flask filled up and siphoned over, carrying its oil extract down to the boiling flask. This process was allowed to go on repeatedly for 4 h before the defatted sample was removed, the solvent recovered, and the oil extract was left in the flask. The flask (containing the oil extract) was dried in the oven at 60 °C for 30 min to remove any residual solvent. It was cooled in desiccator and weighed. The weight of oil (fat) extract was determined by difference and calculated as a percentage of the weight of sample analyzed.

### 2.6 Determination of carbohydrates

The total carbohydrate content was estimated by the method of Hedge and Hofreiter [58], Anthrone Method. Carbohydrate is first hydrolyzed into simple sugars using dilute hydrochloric acid. In hot acidic medium glucose is dehydrated to hydroxymethyl furfural. This compound forms with Anthrone, a green-colored product with absorption maximum at 620 nm. Carbohydrate was determined for the microgreens and sprouts of mung beans. Glucose stock standard was prepared by dissolving 100 mg of glucose in 100 mL of distilled water in a standard flask. Working standard was prepared by pipetting out 10 mL of the stock solution and diluting it to 100 mL such that 1 mL of the working standard contained 100 µg of glucose. 10 mg of the sample was weighed into a boiling tube, hydrolyzed by keeping it in a boiling water bath for 3 h with 0.5 mL of 2.5 N HCl and cooled to room temperature. It was neutralized with solid sodium carbonate until the effervescence ceases and then made up the volume to 100 mL and centrifuged, collected the supernatant and 0.2 mL of it was taken for analysis. Standards were prepared by taking 0.2–1.0 mL of the working standards. 1.0 mL of water serves as a blank made up the volume to 2. 0 mL in all the tubes with distilled water then added 4.0 mL of Anthrone Reagent, heated for 10 minutes in a boiling water bath, cooled rapidly and read the green to dark green color at 620 nm.

Amount of carbohydrates present in 100 mg of the sample = (mg of glucose × 100)/(Volume of test sample).

### 2.7 Determination of crude fiber

Crude fiber was estimated for the microgreens, sprouts and raw pulse of mung beans by the method of James [59], and AOAC 935.14 [60]. Defatted sample (2.0 g) was boiled in 200 mL of 1.25 % H_2_SO_4_ solution for 30 min under reflux. The boiled sample was washed in several portions of hot water using a two-fold cloth to trap the particles. It was returned to the flask and boiled again in 200 mL of 1.25 % NaOH for another 30 min under same condition. After washing in several portion of hot water the sample was allowed to drain dry before being transferred quantitatively to a weighed crucible where it was dried in the oven at 105 °C to a constant weight. It was thereafter taken to a muffle furnace where it was burnt, only ash was left of it. The weight of the fiber was determined by difference and calculated as a percentage of the weight of sample analyzed.

### 2.8 Determination of total ash content

Total ash content was determined using the gravimetric muffle furnace method as described by AOAC 935.14 [60] and James [59]. Approximately 5 g of sample was incinerated in a muffle furnace at 550 °C until complete ashing, cooled in a desiccator, and weighed. Ash content was expressed as a percentage of the sample weight.

### 2.9 Determination of mineral content

Microgreens, sprouts and raw pulse forms of mung beans were analyzed for the probable minerals viz. Ca, K, Mg, Zn, Fe through atomic absorption spectrophotometer (AAS). The test was carried out at Central Instrumentation Laboratory, KVASU, Mannuthy and the equipment used include microwave high pressure digester (PerkinElmer-TITAN 8 – USA, atomic absorption spectrophotometer (PerkinElmer – USA) with graphite furnace or flow injection analysis system (FIAS). Determination of mineral content followed FSSAI [61] method and AOAC 975.03 [62].

Sample preparation was done by weighing 5 g of each sample and drying overnight in the incubator at 4 °C. 6 mL of 70 % nitric acid was pipetted out into Teflon tubes and 1 mL of 30 % hydrogen peroxide and 0.5 g dried sample were added into each of the Teflon tubes. Tightly closed Teflon tubes were kept inside microwave digester for 1 h at 165 °C (30 bar pressure) to complete the digestion. The extracts after digestion were transferred in to labelled test tubes. The extracts were made into suitable dilutions, and the mineral content was determined using AAS with graphite furnace. The mineral content was calculated in mg/L using the formula:

$$\text{Element content }\left(\text{mg}/\text{L}\right)=\text{Concentration }\left(\text{mg}\right)\times \text{volume }\left(\text{V}\right)\times \left(\text{dilution factor}/\text{weight of sample}\right)$$

### 2.10 Estimation of ascorbic acid (vitamin C)

Ascorbic acid content was determined by titrimetric method (AOAC, 967.21) [63] or the microgreens and sprouts of mung beans. Standard stock solution was prepared by dissolving 100 mg of ascorbic acid in 100 mL of 4 % oxalic acid solution in a standard flask (mg/mL). Working standard solution was prepared by pipetting out 10 mL of the stock solution and diluting it to 100 mL using 4 % oxalic acid such that the working standard contained 100 µg/mL. Pipetted out 5 mL of the working standard solution into a 100 mL conical flask and added 10 mL of 4 % oxalic acid which was titrated against the dye (*V*1 mL), end point was the appearance of pink color which persisted for few minutes. The amount of the dye consumed was equivalent to the amount of ascorbic acid.

Then the sample preparation was done by 0.5–5 g depending on the sample) was extracted in 4 % oxalic acid and made up to a known volume (100 mL) and then centrifuged to get the supernatant. Pipetted out 5 mL of this supernatant, added 10 mL of 4 % oxalic acid and titrated against the dye.

$$\text{Amount of the AA mg}/100\text{ mg sample}=\left({0.5/V}_{1}\right)\times \left(V_{2}/5\text{ mL}\right)\times \left(100\text{ mL}/\text{weight of the sample}\right)\times 100$$

*V*_1_ & V_2_ = First and second Titer Values

### 2.11 Determination of total chlorophyll content

Total chlorophyll content was estimated for microgreens and sprouts of mung beans. Chlorophyll was extracted from fresh tissues (about 200 mg) using methanol 99.9 % as solvent. Samples were kept in a dark room at 4 °C for 24 h. Quantitative chlorophyll determinations were carried out immediately after extraction. Absorbance readings were measured at 663 and 645 nm for the calculation of chlorophyll *a* (Chl. a) and b (Chl. b), respectively. Chlorophyll concentrations were calculated by Lichtenthaler’s formula (1987) [64].

$$\text{Chlorophyll }a=\left(12.7\times \text{OD at }663\text{ nm}\right)-\left(2.69\times \text{at }645\text{ nm}\right)$$

$$\text{Chlorophyll }b=\left(22.9\times \text{OD at }645\text{ nm}\right)-\left(4.08\times \text{OD at }663\text{ nm}\right)$$

Total cholorophyll=chlorophyll a+chlorophyll b

### 2.12 Microbial analysis

The samples for standard plate count (SPC) were prepared as referred to AOAC 966.23 [65]. The incubation of the prepared plates was done in an inverted position at 35 °C ± 2 °C for 48 h. Following incubation, all colonies on dishes containing 30–300 colonies were counted and the results were recorded per dilution.

$$\text{Number of colonies}=\left(\text{Average number of colonies}\right)/\left(\text{Amount plates}\times \text{dilution}\right)$$

The count expressed in CFU/mL=number of colonies×dilution factor

Enumeration of yeast and mold performed using PDA for an incubation period of 5–7 days at 20 or 25 °C. The colonies were counted and multiplied by the inverse of the corresponding dilution and reported as yeast or mold count per gram. Microbial counts (Colony Forming Unit (CFU)/mL) were log-transformed (log_10_) prior to statistical analysis to normalize data distribution and stabilize variance.

### 2.13 Pathogen isolation

*Salmonella* and *Shigella* – A sample of 25 g was weighed and made into 1:10 dilution using peptone water and incubated overnight for growth. From the previously inoculated culture, 1 mL was inoculated to 9 mL tetrathionate broth and incubated overnight. Sub culturing was done by taking 1 mL of this culture and pour plating to bismuth sulphate agar (BSA) or Salmonella/Shigella agar and incubation was carried out for overnight. Formation of typical black colonies with green metallic sheen indicates the presence of Salmonella and/or Shigella [66].

Coliforms – A sample of 25 g was made into 1:10 dilution (into 250 mL) using saline water and incubated overnight for growth. From the previously inoculated culture, 1 mL was inoculated to 9 mL MacConkey broth and incubated overnight. Subculturing was done by taking 1 mL each of this culture and pour plating into Eosine Methylene Blue (EMB) agar and Violet Red Bile Agar (VRBA) to detect the presence of coliforms [66].

### 2.14 Statistical analysis

Data were expressed as mean ± standard deviation (SD) based on duplicate measurements (*n* = 2). Statistical analysis was performed using one-way analysis of variance (ANOVA), and significant differences among the means were determined at a significance level of *p* < 0.05. Means followed by different letters indicate statistically significant differences according to Tukey’s post hoc test.

## 3 Results

### 3.1 Awareness and perception of microgreens

**Figure 1:**
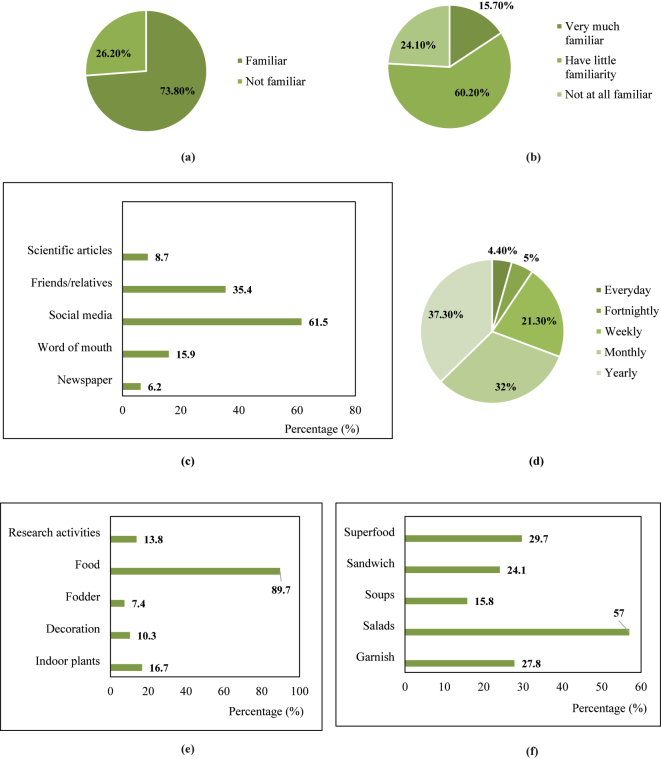
(a), (b). Familiarity of people surveyed with microgreens; Figure 1, (c) source of information: microgreens; Figure 1, (d) frequency of consumption; Figure 1, (e), (f). Common use of microgreens

An online survey was conducted among the general public to assess awareness and perceptions of microgreens, yielding a total of 229 valid responses. The majority of respondents were aged 18–35, with Indian citizens representing the largest proportion. Responses from other continents, including Europe and Australia, were limited. A graphical representation of the responses was accessed from the Google Forms. Among them, 73.8 % were familiar with the word ‘microgreen’ (Figure 1(a)). 15.7 % of the respondents were highly familiar with microgreens, 24.1 % had limited familiarity, while 60.2 % had no prior knowledge (Figure 1(b)). Perceptions varied: some identified microgreens as nutrient-dense supplements or visual and flavor enhancers, while others defined them as young vegetable greens harvested post-cotyledon stage. Among those aware, 61.5 % learned about microgreens via social media, followed by friends/relatives (35.4 %), word of mouth (15.9 %), research publications (8.7 %), and newspapers (6.2 %) (Figure 1(c)).

Survey findings indicated that 89.7 % of respondents reported consuming microgreens, while 19.5 % had experience growing them. Despite high awareness, primarily through social media, only 16.7 % recognized their potential as indoor plants. A minority associated microgreens with research use (13.8 %), decoration (10.3 %), or fodder (7.4 %) (Figure 1(e)). Regarding consumption frequency, 37.3 % consumed them annually, 32 % monthly, and the rest weekly, fortnightly, or daily (Figure 1(d)). The most common uses were in salads (57 %), followed by garnishing (29.7 %), soups, and sandwiches (Figure 1(f)). Although 93.9 % acknowledged their health benefits and expressed interest in future use, actual cultivation and regular consumption remain limited, likely due to availability issues, cultivation challenges, and unfamiliarity with the product. The observations from the survey conducted depicted that many people are increasingly interested in enhancing the nutritional quality of the foods they consume, and hence are ready to adopt the innovations soon.

### 3.2 Development of sprouts and microgreens

**Figure 2:**
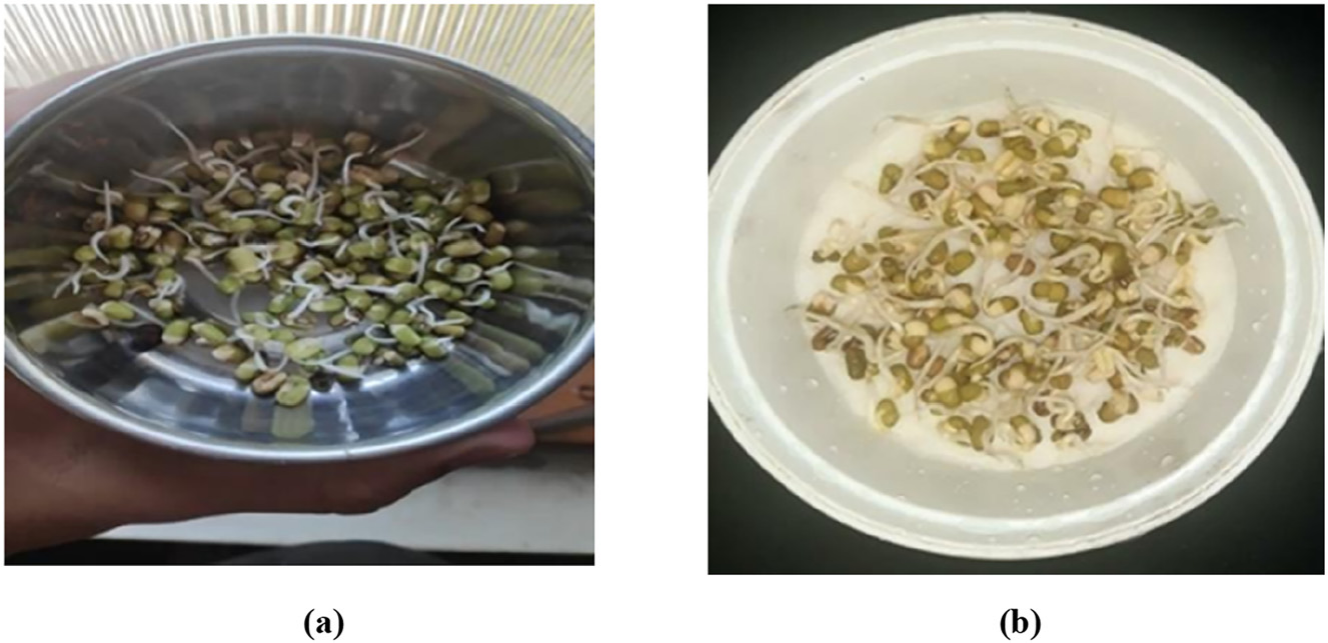
(a), (b). Production of mung bean sprouts (photo: Marottickal).

Sprouting initiated within 1–2 days post-soaking (Figure 2), with the resultant sprouts reaching lengths between 6 and 9 mm, consistent with the observations reported by Ketaki and Sasikala [67].

**Figure 3:**
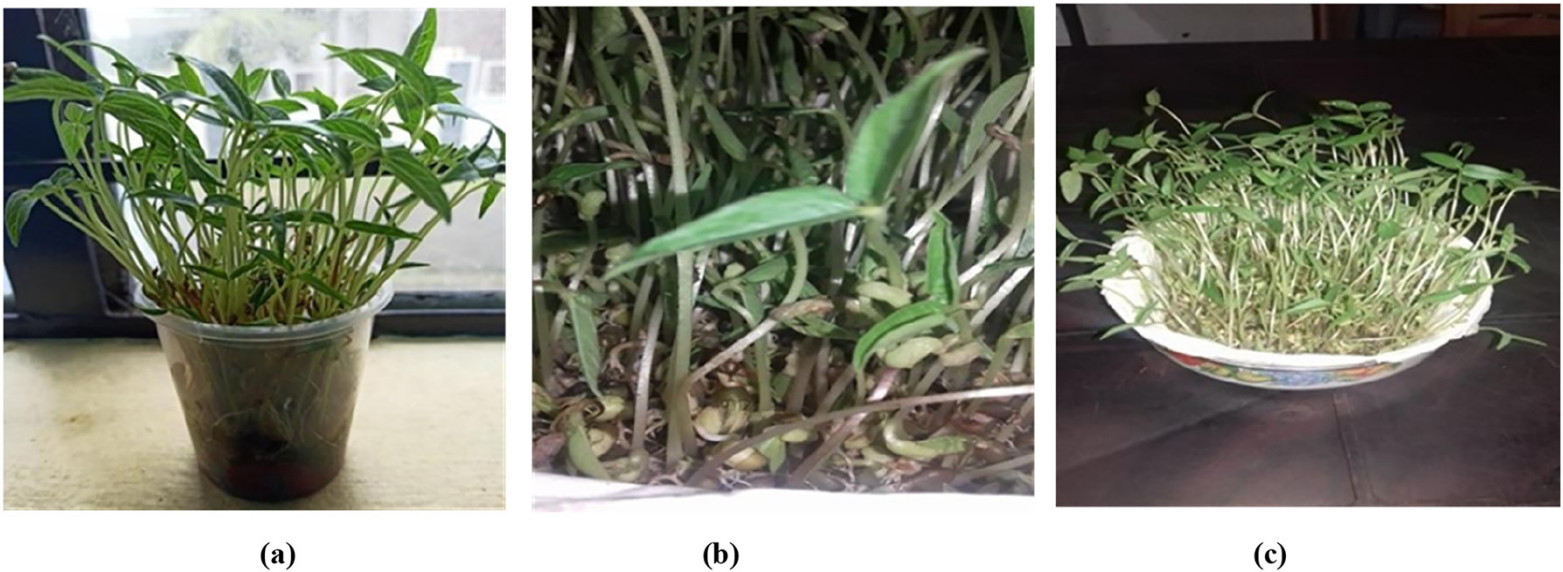
(a), (b), (c). Production mung bean microgreens (photo: Marottickal).

Microgreens started growing from sprouts as roots became more fibrous and true leaves emerged. The sprouts were grown into microgreens within 4–5 days after the germination of seeds. It took 7 days for the appearance of the first pair of true leaves (Figure 3). The samples were harvested for analysis in 4–5 days after germination. The length of the microgreens in 5 days was in the range of 10–12 cm, and that in 7 days was in the range of 13–16 cm. The leaves exhibited a size between 5 and 10 mm (Figure 3(b)). Both sprouts and microgreens were cultivated in parallel under controlled environmental conditions (26–28 °C, 80 % relative humidity, 0.997–0.999 atm) to minimize potential environmental bias.

Danielle et al. [68] reported that microgreens are typically harvested at the first true leaf stage, at an average height of approximately 5.08 cm, with the duration from seeding to harvest varying among crops from 7 to 21 days. In general, microgreens are harvested when plant height ranges between 2.54 and 7.62 cm, depending on the species and growth conditions [14].

### 3.3 Proximate composition

The proximate analysis for the microgreens (MG), sprouts (SP) and raw pulse (RP) was estimated, and the results are given in Table 1. The nutrients were present in all three growth stages of mung beans in variable levels.

**Table 1:** Proximate composition of MG, SP and RP of mung beans.

SI no	Parameters	MG	SP	RP
1	Moisture (%)	85.144 ± 0.014^a^	62.259 ± 0.013^b^	11.849 ± 0.077^c^
2	Total solids (%)	14.856 ± 0.014^c^	37.741 ± 0.013^b^	88.151 ± 0.077^a^
3	Crude protein (%)	45.840 ± 0.010^a^	31.810 ± 0.010^b^	15.870 ± 0.027^c^
4	Crude fat (%)	2.693 ± 0.094^b^	2.940 ± 0.056^a^	2.300 ± 0.016^c^
5	Carbohydrate (%)	53.110 ± 0.034^a^	126.1 ± 2.4^b^	–
6	Crude fiber (%)	52.640 ± 0.027^a^	13.160 ± 0.280^b^	4.480 ± 0.160^c^
7	Total ash (%)	3.560 ± 0.094^b^	3.250 ± 0.034^c^	5.210 ± 0.130^a^

Values are expressed as mean ± STDEV (standard deviation) (*n* = 3) on dry matter basis; Letters (a–c) indicate a significant difference at *p* < 0.05 on one-way Anova; DM values >100 % indicate carbohydrate concentration relative to the dry fraction of the sample.

Moisture content decreased from MG (85.14 %) to RP (11.85 %), with a corresponding increase in total solids from 14.86 % to 88.15 %. Crude protein was highest in MG (45.84 %) and lowest in RP (15.87 %), whereas crude fat content was comparable among samples, with SP showing a slightly higher value (2.94 %). Carbohydrate content obtained by calculation method was 53.11 % in MG, while on a dry matter basis, SP exhibited 126.1 %, reflecting the concentration effect after water removal. Values above 100% reflect methodological artifacts of the difference approach and should be interpreted comparatively rather than as absolute percentages. Crude fibre was predominant in MG (52.64 %), and total ash content was highest in RP (5.21 %). These results indicate distinct compositional differences among the samples, driven largely by moisture and dry matter distribution.

### 3.4 Glucose concentration

**Figure 4:**
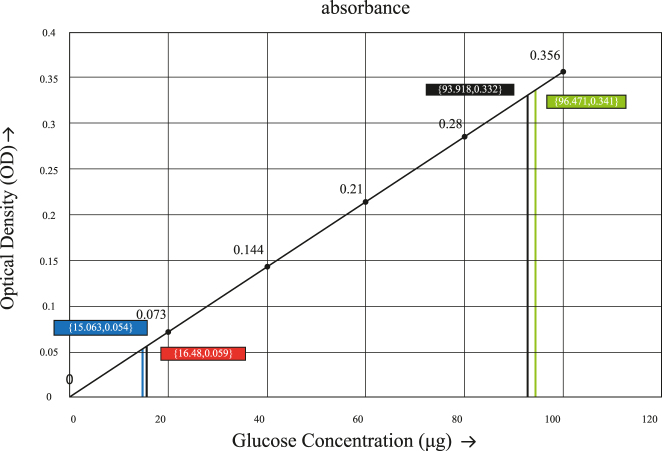
Standard glucose curve with the values of glucose concentrations of MG and SP of mung beans in 0.2 ml of the sample.

Figure 4 illustrates the glucose standard calibration curve, constructed by plotting known glucose concentrations (µg) on the *x*-axis against their corresponding optical density (OD) readings on the *y*-axis. The resulting regression equation was *y* = 0.00354*x* + 0.00225, *R* ^2^ = 1.

### 3.5 Mineral content

**Figure 5:**
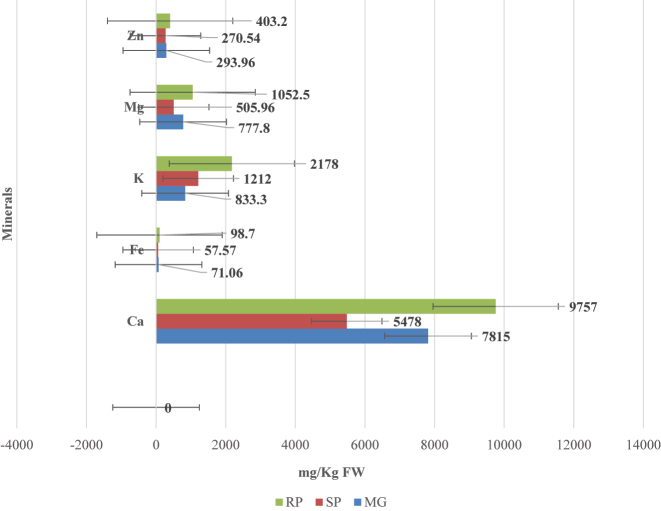
Mineral content of MG, SP and RP of mung beans analyzed through AAS; values are expressed as mean (*n* = 3).

The results of the mineral content analyzed per kg fresh weight (FW) are given in the Figure 5.

The raw powder (RP) consistently shows the highest concentration of all measured minerals (Calcium, Iron, Potassium, Magnesium, and Zinc), indicating nutrient densification likely due to moisture removal. In contrast, microgreens (MG) and sprouts (SP) contain lower mineral levels, with SP generally having intermediate values between MG and RP.

### 3.6 Ascorbic acid (vitamin C)

The ascorbic acid content estimated for the SP and MG of mung beans are given in Table 2. Microgreens contain significantly higher levels of ascorbic acid (110.96 mg/100 g) compared to sprouts (88.89 mg/100 g), indicating superior vitamin C content. This suggests that microgreens may offer greater antioxidant potential and nutritional benefits in terms of ascorbic acid concentration.

**Table 2:** Ascorbic acid content of the MG and SP.

SI No	Parameter	Microgreens	Sprouts
1	Ascorbic acid (mg/100 g)	110.955 ± 0.063^a^	88.888 ± 0.024^b^

Values were expressed as mean ± STDEV (standard deviation) (*n* = 3); Letters (a–b) indicates significant difference at *p* < 0.05.

### 3.7 Total chlorophyll content

Microgreens exhibit higher concentrations of chlorophyll *a* (0.037 mg/g), chlorophyll *b* (0.005 mg/g), and total chlorophyll (0.0425 mg/g) compared to sprouts (*p* > 0.05), which have lower values across all parameters, when analysed on a fresh-weight basis. This indicates that microgreens possess greater photosynthetic pigment content, potentially enhancing their antioxidant and nutritional properties.

### 3.8 Enumeration of microbes

The samples were used unwashed to assess the “as-consumed” microbial quality, reflecting what consumers would actually encounter rather than sterile conditions. Sprouts (SP) exhibit higher microbial loads across all tested parameters, including aerobic mesophilic count, coliforms, *Escherichia coli*, and yeast and mold, compared to microgreens (MG), where *E. coli* was notably absent.

## 4 Discussion

### 4.1 Moisture content

The moisture content was significantly higher (*p* < 0.05) in MG (85.144 ± 0.014 %) compared to SP (62.259 ± 0.013 %) and RP (11.849 ± 0.077 %) of mung beans (Table 1). This high-water content promotes susceptibility to microbial growth and enzyme activities. This indicates a reduced shelflife for MG when compared to its SP and RP. According to Berba and Uchanski [69], use of MG is limited by an extremely short shelf life of approximately one week, depending on the species. This perishability impedes further market growth and export of the crop. In contrast, sprouts are typically harvested with the roots intact, which allows for a longer shelf life. Hence, perishability becomes the major concern for marketing. The moisture content of mung bean SP was in support of the scientific findings [70]. According to Wojdylo et al. [44], high content of water in microgreens and some sprouts (black medick, sunflower, beetroot and mung bean) indicated high water storage capacity and adaptations preventing water loss.

### 4.2 Total solids

Total solids were determined as the complement of moisture content. As anticipated, RP exhibited the highest total solids, followed by SP and MG, reflecting their progressively lower moisture levels. Although all proximate composition parameters were initially measured on a fresh weight basis, expressing the data on a dry matter basis is essential to avoid overestimation of nutrient density in dried samples and underestimation in fresh tissues (Table 1). This normalization facilitates more accurate and meaningful comparisons of nutrient composition among samples with markedly different water contents.

### 4.3 Crude protein

Crude protein content differed markedly among the samples, with MG exhibiting the highest value and RP the lowest. Such variation in crude protein concentration is commonly observed in proximate composition studies and is influenced by intrinsic factors such as species/varietal differences and biochemical composition, as well as environmental and physiological conditions affecting nitrogen and protein synthesis pathways in biological materials [71]. Table 1 shows highest protein content in MG followed by SP and RP, in dry matter basis. This is line with studies of Kajszczak et al. [72] in radish sprouts, where the protein fraction was greater than in radish microgreens, indicating that the sprouting stage can be associated with elevated protein levels relative to later microgreen stages. The protein content in the microgreens of mung beans was significantly higher when compared to the microgreens of other seed varieties viz Brassicaceae, Asteraceae microgreens as reported in Paradiso et al. [73]. According to Rusydi et al. [70], protein content was significantly decreased in germinated legume. Vellupillai et al. [74] observed that the decrease in total protein content was simultaneous with increase in amino acid content caused by increased level of protease activity. Furthermore, the drop in protein content seems to indicate that proteolysis outpaces protein synthesis in the growing sprouts [75].

### 4.4 Crude fat

The observed differences in crude fat content among the samples likely reflect inherent compositional and biological variation as well as sample-specific factors. As shown in Table 1, the crude fat content decreased in RP while SP with the highest value. The results obtained were in line with the results of Marton et al. [76], Shah et al. [77] and Paradiso et al. [73], as they reported a significant reduction in fat content in the case of MG. The general reduction in fat content might be due to the utilization of the fat as energy source for the growth of the MG or due to the loss of total solids during germination as well as its further growth into MG [77]. The fat content of SP obtained from this study was comparatively lower to the results of Rusydi et al. [70].

### 4.5 Glucose concentration and carbohydrate content

The glucose standard curve generated in this study (Figure 5) displayed a strong linear relationship between glucose concentration and absorbance, consistent with the expected behavior of the Anthrone reaction as described in classical carbohydrate assay literature [78]. The steadily increasing absorbance values confirm proper dehydration of carbohydrates to furfural derivatives, followed by stable chromogen formation with the Anthrone reagent, a reaction mechanism well-documented in biochemical analyses [79].

The high linearity of the regression line confirms the reliability and accuracy of the assay method. This standard curve was subsequently employed to estimate the glucose content in unknown samples by interpolating their OD values. Vertical lines in the graph represent the extrapolated concentrations from specific OD readings. For the MG sample, lower OD values of 0.054 and 0.059 corresponded to glucose concentrations of 15.063 µg and 16.48 µg, respectively. In contrast, the SP sample showed higher OD values of 0.332 and 0.341, translating to significantly elevated glucose concentrations of 93.918 µg and 96.471 µg, respectively. These results align with the percentage carbohydrate content trajectory, wherein SP exhibited a higher glucose concentration than MG, reflecting its greater carbohydrate content. This comparative quantification underscores the effectiveness of the colorimetric assay in differentiating the glucose levels across diverse sample matrices.

In the case of carbohydrate content, the estimation based on difference method rather than direct quantification influenced their apparent distribution among the samples. In the case of raw pulses (RP), the relatively high contributions of protein, crude fiber, ash, and total solids resulted in negligible residual values when carbohydrates were calculated by difference, leading to the absence of a reported value. This does not indicate a true lack of carbohydrates in raw pulses, as these are known to contain substantial amounts of starch and structural polysaccharides that serve as primary energy reserves for germination. Instead, this outcome highlights a limitation of indirect carbohydrate estimation methods, particularly in samples with high levels of other proximate components [80].

According to Rusydi et al. [70], carbohydrate content increases in germinated mung bean. In contrast, the present study observed approximately a 2.4-fold reduction in carbohydrate content in the MG following germination. This decrease is consistent with the findings of Vidal-Valverde et al. [81], who reported that carbohydrates are mobilized as an energy source to support embryonic growth during germination, explaining the observed reduction. As shown in Table 1, this decrease was statistically significant (*p* < 0.05) compared to the sprout (SP), highlighting the potential of mung bean MG as a suitable option for low-calorie diets. This decrease in carbohydrate might be due to the breaking down of starch in cotyledon into smaller molecules such as glucose and fructose to provide energy for cell division while the seeds mature and grow [81], 82]. Ohtsubo et al. [83] explained that carbohydrate breakdown in which α-amylase activities were found to parallel with the pattern of starch breakdown. Additionally, sprout and microgreen leaves are characterized by a low calorific value (29–128 kcal/100 g) and low glycemic index [35], 46]. This trend of reduced carbohydrate content in MG compared to SP is consistent with the glucose concentration results depicted in Figure 5, where SP exhibited markedly higher glucose concentrations than MG. These findings should be interpreted in light of the small sample size (*n* = 2) and the use of indirect carbohydrate estimation, which may introduce additional variability.

### 4.6 Crude fibre

According to Di Gioia and Santamaria [84] and Paradiso et al. [73], the crude fiber content in the Brassica microgreens showed lower levels. Total dietary fibers were significantly decreased in germinated mung beans [70]. In contrast, the crude fiber obtained from this study for the mung bean SP (13.160 ± 0.280 per cent) and MG (52.640 ± 0.027 per cent) were much higher than values reported for Brassica microgreens. Furthermore, the increase in crude fiber content from SP to MG were significant (*p* < 0.05) (Table 1). The higher fiber content may improve satiety and support digestive health. Marero et al. [85] reported that the effect of germination on fiber was dependent on type of legumes. The increased fiber content may be due to the variety of the mung beans used for the study. A fiber-rich diet is lower in energy density, often has a lower fat content, is larger in volume and is richer in micronutrients [86].

It is suggested that healthy adults should eat between 20 and 35 g of dietary fiber each day. Several non-starch foods provide up to 20–35 g of fiber/100 g dry weight and other those containing starch provide about 10 g/100 g of dry weight and the content of fiber of fruits and vegetables is 1.5–2.5 g/100 g of dry weight [59]. Since the crude fiber obtained from thus study was much higher as compared to that reported by Selvendran and Robertson [87] for the fruits and vegetables, the MG as well as SP form a good source of fiber in the diets. These features make mung bean microgreens well suited as toppings for salads, sandwiches, and other ready-to-eat dishes where small portions can still contribute meaningfully to fiber and mineral intake.

### 4.7 Total ash content

The total ash content varied significantly among the samples (*p* < 0.05), with RP exhibiting the highest mineral content (5.21 %), followed by MG (3.56 %) and SP (3.25 %), reflecting differences in inorganic nutrient accumulation across the sample types. The ash content obtained was calculated on moisture free basis. The decrease in ash content in SP and MG might be due to the loss of carbohydrates, fat, and other solids during the process of microgreen development. Wang et al. [88] reported that as the soaking time increase there is reduction in minerals as the seed utilizes then for emergence of rootlet. Shah et al. [77] reported a decrease in ash content in certain varieties of mung beans as the seed grown into microgreens (in the case of Ramzan variety). According to Butkutė et al. [89], the ash content varies depending on the microgreen varieties, and it was reported that the ash content increased as the RP germinated to SP but decreased as it grown into MG. The ash content of mung bean SP decreased significantly from its RP [70]. The total ash content obtained from this was comparable to the results obtained by Wojdylo et al. [44].

### 4.8 Mineral content

For mineral content, a tremendous increase was observed in its quantity from SP to MG. (Figure 4). At the same time, RP has high amount of the mentioned minerals than other forms of the pulse. Pinto et al. [20] has conducted studies which are in line with the obtained results. Their results showed that microgreens possess a higher content of most minerals including Ca, Mg, Fe, Mn, Zn and Mo than other vegetables [90]. Ca and Mg content were in high amount in MG compared to SP. As the plant develops from the seed, leaf formation occurs which lead to increase in chlorophyll thereby magnesium. On comparison with studies conducted by Xiao [91] and Weber [90], that showing increased K content in microgreens, the results obtained from this study showed a decrease in K which is a contradiction. Even though selected minerals were present in higher content in RP, its bioavailability enhances as it become SP and MG.

Microgreens have been reported to contain higher concentrations of mineral compounds (such as Ca, Mg, Fe, Mn, Zn, Se, and Mo) and phytonutrients (including ascorbic acid, β-carotene, α-tocopherol, and phylloquinone) compared to their mature-leaf counterparts. Owing to these enhanced nutritional properties, microgreens represent a valuable dietary source for individuals with elevated nutritional needs, particularly vegetarians and vegans, who can incorporate a diverse range of microgreens to enrich their diets. Furthermore, as microgreens are generally consumed raw, they are also well-suited to meet the dietary preferences of raw food enthusiasts [92].

### 4.9 Ascorbic acid (vitamin C)

The SP and MG samples were analysed for ascorbic acid (AA) content. The results showed the values of 110.955 ± 0.063 mg/100 g and 88.888 ± 0.024 mg/100 g for MG and SP respectively. AA is practically absent in dry legume seeds, increases in significant amount when developed into sprouts and microgreens as reported by Shah et al. (2011) which is well in agreement with the results obtained from this study. According to Mao-jun et al. [93], the AA content varies with fluorescence, species, germination time etc. The AA content obtained from this study for the microgreens and sprouts of mung beans was in line with the results obtained by Thippeswamy et al. [94]. The study done by Xiao [22] also supported the results from this study as it reported that, the total AA content for 25 varieties of microgreens studied, ranged from 20.4 to 147.0 mg/100 g FW. The AA can be oxidized into dehydroascorbic acid (DHA) when the plant is subject to physical or physiological stress (harvesting injury, chilling, irradiation, etc.) [95]. A significant difference (*p* < 0.05) was observed in the results of MG (110.955 ± 0.063 mg/100 g) and SP (88.888 ± 0.024 mg/100 g), making mung bean MG a rich source of AA when compared to its SP (Table 2).

### 4.10 Total chlorophyll content

The total chlorophyll content in mung bean microgreens (MG) observed in this study was comparable to that reported for commercially sourced broccoli microgreens by Tan et al. [96]. A slight reduction in chlorophyll content was noted relative to farm-sourced broccoli MG in the same study. However, these findings contrast with those of Kumar et al. [96], who reported a significantly higher chlorophyll content of 4.07 mg g^−1^. Microgreens showed numerically higher chlorophyll a, b, and total chlorophyll than sprouts, although differences were not statistically significant (*p* > 0.05; Table 3).

**Table 3:** Total chlorophyll content of MG and SP of mung beans.

S. No.	Sample	^a^Chlorophyll *a*	^a^Chlorophyll *b*	^b^Total chlorophyll content
1.	Microgreens	0.037 ± 0.005	0.005 ± 0.001	0.0425 ± 0.009^a^
2.	Sprouts	0.013 ± 0.002	0.008 ± 0.001	0.0203 ± 0.001^a^

^b^Expressed in mg/g; Values were expressed as mean ± STDEV (*n* = 3); Letter (a–a) indicates no significant difference at *p* < 0.05, calculated for total chlorophyll. The samples were analyzed in FW.

Recently, chlorophyll and chlorophyll-rich diets have been reported to play roles as cancer-preventive agent attributed to the ability of chlorophyll to form complexes with specific carcinogens, as well as its antioxidant and anti-mutagenic properties [97], 98]. Some other preventive or therapeutic properties of chlorophyll were also reported in literature, such as stimulating immune system, detoxification of the liver, and normalizing blood pressure [99], 100].

### 4.11 Pathogen isolation and microbial safety

Several pre-harvest and post-harvest factors may cause the microbial build up on both the sprouts and microgreens including the tissue paper (substratum), humidity, temperature, and wash-treatments adopted. Total aerobic mesophilic bacteria (AMB) plate count for SP and MG is 7.012 and 6.089 respectively (Table 4), which are considerably high and comparable to that reported for cilantro and baby spinach [41], 42], 100], 101]. It has been hypothesized that delicate, soft textured hypocotyls of sprouts and microgreens may favor microbial growth compared to RP and mature counterparts [41].

**Table 4:** Microbial count using TPC and Pour plating, for unwashed samples.

SI. No.	Parameters	SP (log CFU/g)	MG (log CFU/g)
1	Aerobic mesophilic count (AMB)	7.012	6.089
2	Coliform	9.152	8.595
3	*E. coli*	7.117	No colonies observed
4	Yeast and mold	7.012	5.812

Yeast and mold plating was carried out using PDA with 1 % of 10 % citric acid and the population were 7.012 log CFU/g and 5.812 log CFU/g in SP and MG respectively, which are higher than that reported in Tournas [102], where the most prevalent organisms found in the samples including 39 ready-to-eat salads, 29 whole fresh vegetables and 116 sprout samples under experimentations. The yeast count ranges from less than 100 to 4.0 × 108 CFU/g and mold count range from less than 100 to 4.0 × 10.4 CFU/g. During the study, the count observed were high in microgreen root environment or samples that having more root parts which was relatable with the studies conducted by Gina Marie Misra [103], on food safety risk in an indoor microgreen cultivation system.

On a regulatory forefront, Codex Alimentarius does not currently provide universal numeric limits for microbial indicators specifically for sprouts or microgreens, leaving the establishment of microbiological thresholds to national authorities or commodity-specific standards. In relation to EU standards, the microgreen samples produced in the study were deemed suitable for human consumption in their unwashed form as they are categorized under the general fresh-produce hygiene framework. This assessment is further supported by the absence of *E. coli*, indicating compliance with the EU microbiological criteria for sprouted seeds [104] as well as relevant Indian standards [105]. Given that the study was conducted exclusively within an Indian context, the primary reference for regulatory analysis was the Food Safety and Standards Authority of India (FSSAI). According to FSSAI (2023) [105], process hygiene standards for fresh vegetables do not define specific numeric limits for microbial indicators. Within this framework, sprouts and microgreens are treated as fresh vegetables or minimally processed produce, and their microbiological safety is assessed primarily through hygiene practices and pathogen absence rather than quantitative limits for aerobic plate count, coliforms, or yeasts and moulds.

*Salmonella* and *Shigella* were not detected in both the MG as well as SP of mung beans. The coliform and *E coli* counts were comparatively higher for SP than MG (Table 4). The counts were comparable to the studies conducted by Xiao et al. [106]. This indicates that as the SP grows into MG the microbial load decreased. Seeds have been found to be the main source of food borne illness outbreaks [107], 108]. Therefore, obtaining seeds that have been certified for sprouting and produced using good agricultural practices is an important step in microgreen production. This is not very surprising, from a micro ecological standpoint, production of sprouts and microgreens differ in a number of operating steps: Microgreens undergo a brief seed-soaking period, are generally grown on substrate, and are consumed after cutting without the root system. On the other hand, sprouts are eaten raw together with their roots. Thus, many food agencies classify sprouts as “high risk” products, while microgreens are considered much safer, although some (low) risks can arise from fresh herbs (basil, thyme) or baby-leaf vegetables (baby spinach) [109], [110], [111]. Berba et al. [69] also states that particular condition of microgreen growth reduces microbial load to a safer level ensuring quality. However, negative controls were not included in this study, which limits the ability to account for background contamination during sample handling or analysis. Therefore, it is hard to claim the safety of the final fresh produce, as in overall. From a consumer perspective, thorough washing and good seed hygiene remain essential to minimize risk, particularly for sprouts.

## 5 Conclusions

The present study evaluated the proximate and mineral composition of a locally available mung bean variety and demonstrated that microgreens and sprouts share comparable nutritional characteristics, with distinct differences in specific components. The results showed that mung bean microgreens possessed a higher moisture content, whereas sprouts exhibited slightly higher fat content and total ash than microgreens, while crude protein was highest in microgreens. In contrast, microgreens were characterized by significantly higher dietary fiber levels, lower fat and carbohydrate contents, and enhanced concentrations of essential minerals, including calcium (Ca), magnesium (Mg), zinc (Zn), and iron (Fe).

The elevated fiber and mineral contents highlight the nutritional potential of mung bean microgreens as a complementary component of the human diet. Overall, the findings support the inclusion of mung bean microgreens as a nutritionally valuable and sustainable food option. However, considering the potential microbial safety concerns associated with the consumption of raw microgreens and sprouts, appropriate handling and processing practices are necessary. Future studies should extend this work to microgreens from diverse plant species, with an emphasis on comprehensive nutritional evaluation and value-added applications.
